# Climate and symbioses with ants modulate leaf/stem scaling in epiphytes

**DOI:** 10.1038/s41598-019-39853-4

**Published:** 2019-02-22

**Authors:** Guillaume Chomicki, Susanne S. Renner

**Affiliations:** 1 Department of Plant Sciences, University of Oxford, South Park Road, Oxford, OX1 3RB UK; 2 The Queen’s college, High St, Oxford, OX1 4AW UK; 3 Systematic Botany and Mycology, Department of Biology, University of Munich (LMU), Menzinger Str. 67, 80638 Munich, Germany

## Abstract

In most seed plants, leaf size is isometrically related to stem cross-sectional area, a relationship referred to as Corner’s rule. When stems or leaves acquire a new function, for instance in ant-plant species with hollow stems occupied by ants, their scaling is expected to change. Here we use a lineage of epiphytic ant-plants to test how the evolution of ant-nesting structures in species with different levels of symbiotic dependence has impacted leaf/stem scaling. We expected that leaf size would correlate mostly with climate, while stem diameter would change with domatium evolution. Using a trait dataset from 286 herbarium specimens, field and greenhouse observations, climatic data, and a range of phylogenetic-comparative analyses, we detected significant shifts in leaf/stem scaling, mirroring the evolution of specialized symbioses. Our analyses support both predictions, namely that stem diameter change is tied to symbiosis evolution (ant-nesting structures), while leaf size is independently correlated with rainfall variables. Our study highlights how independent and divergent selective pressures can alter allometry. Because shifts in scaling relationships can impact the costs and benefits of mutualisms, studying allometry in mutualistic interactions may shed unexpected light on the stability of cooperation among species.

## Introduction

Traits that scale with body size can show different patterns across developmental stages, between individuals, and among species, and their covariation is frequently distinct from isometry and sometimes even nonlinear^1–3^. In the strict sense, any deviation from isometry is referred to as allometry. Three levels of allometry are commonly distinguished: ontogenetic allometry, referring to size covariation among traits during growth; static allometry, referring to trait covariation among individuals of a species at the same developmental stage; and evolutionary allometry, referring to trait covariation among different species at the same developmental stage^4,5^. These three levels of allometry are often related, at least in animals^5,6^. While many allometric patterns have been documented, little is known about the possible ecological context of shifts in allometries. Allometry was initially considered to arise from developmental constraints^2,7^, but more recent work has favoured a role of natural selection^3,8^, although constraint and selection are hard to disentangle^9^. Work on insects has shown that allometric slopes can evolve independently of mean trait values^10^ and that even highly conserved relationships can show lability^11^, highlighting that natural selection, for instance, under changing ecological conditions, can bypass developmental constraints.

Body parts of vascular plants – leaves, shoots, branch systems – are linked to one another, and evolutionary change in one impacts the others. Corner^12^ was the first to note that appendage size is isometrically related to the diameter of the (primary) stem bearing the appendages (such as leaves), an empirical pattern known as Corner’s rule^13^. This rule has been verified in many species of trees^14–18^, and a meta-analysis 20 years ago confirmed that the isometric relationship between twig cross-sectional area (before secondary growth) and the surface of a fully expanded leaf is conserved in deciduous angiosperms, evergreen angiosperms, and gymnosperms, but with distinct intercepts^19^. This suggests that stem vascular supply is proportional to the demands of the supported leaves^19^. Factors changing this trade-off are therefore expected to affect leaf/stem scaling. This is the case in lianas, where the loss of the self-supporting function of the stem leads to significantly larger leaf biomass for the same stem diameter compared to trees^20^.

Another new function of stems is that they can form cavities (domatia) adapted for housing ant symbionts. Domatia involving hollow stems are expected to change stem/leaf scaling because of the extra mechanical support needed to sustain a hollow stem^19,21,22^. Such allometric change has indeed been found in three ant-plant lineages, namely *Macaranga* (Euphorbiaceae), *Leonardoxa* (Fabaceae) and *Triplaris* (Polygonaceae)^21,22^. Overall, nearly 700 plant species form symbioses with ants, offering nesting sites in modified stems or leaves, in return for protection and/or nutrition^23^.

**Figure 1:**
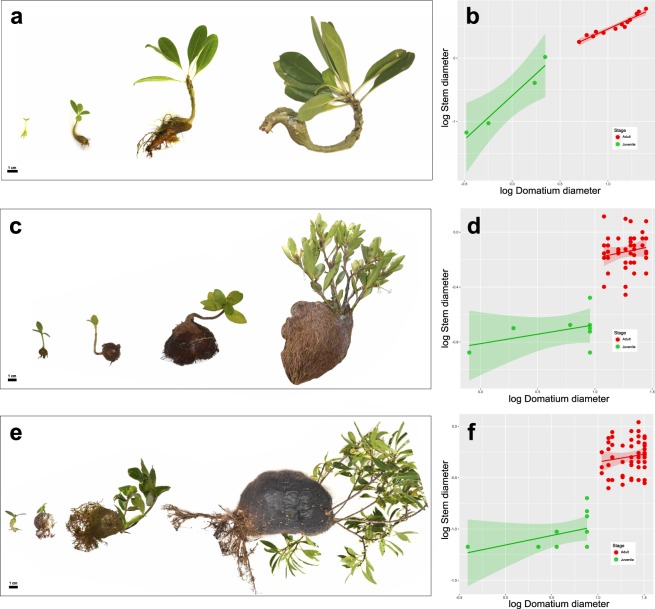
Architecture and domatium development in the Hydnophytinae (Rubiaceae). Developmental stages and domatium/stem scaling in the Hydnophytinae (**a**,**b**), *Myrmecodia tuberosa*, a specialized ant-plant. Note that in (**b**), domatium and stem scaling are correlated throughout the plant’s development, meaning that the (single) stem and (always single) domatium grow jointly. The domatium grows only apically. (**c,d**) *Squamellaria wilkinsonii*, a generalist ant-plant. Multiple stems originate from the domatium apex throughout the plant’s life (**c**) and are thus not correlated with domatium development. The domatium grows diffusely. (**e,f**) *Squamellaria wilsonii*, a specialized ant-plant with multi-stemmed architecture and an apically growing domatium. As in *S. wilkinsonii*, stem and domatium growth are only loosely correlated as new stems develop throughout the plant’s life, but as in *Myrmecodia*, the domatium grows apically, not diffusely. In the right panel, each point represents a stem associated with a domatium, meaning that in (**b**) each point represents an individual, and in (**d**) and (**f**) for a given x-axis position, the  data points  represents the distincts stems belonging to  the same individual plant  (since these two species are multi-stemmed).

**Figure 2:**
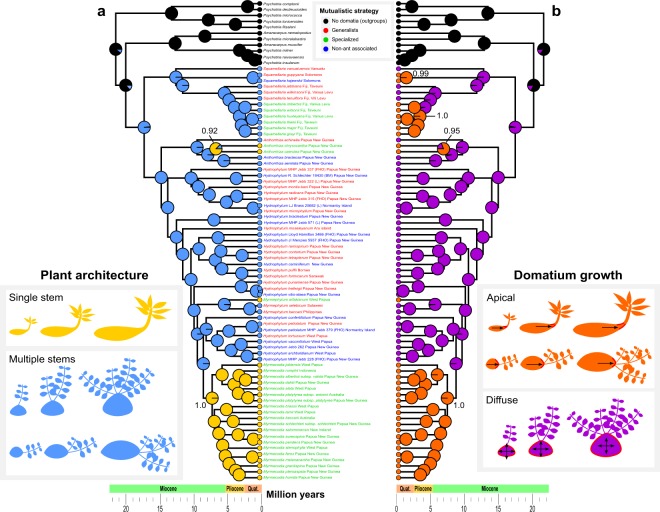
Ancestral state reconstruction of plant architecture (multi-stem versus single-stem) (**a**) and domatium growth type (diffuse or apical) (**b**), from 1,000 simulations of character states on a dated phylogeny for 70% of all species of Hydnophytinae and a reverse-jump MCMC approach on 1,000 trees (probability shown at key nodes). In (**a**), the lower panel shows the two types of multi-stem architecture, in species with domatium growing diffusely as in *S. wilkinsonii* [Fig. 1c,d] (top) or apically as in *S. wilsonii* [Fig. 1e,f] (bottom). In (**b**), the top panel shows two types of apical domatium growth: associated with single-stemmed architecture as in *Myrmecodia* [Fig. 1a,b] (top) or associated with multi-stem architecture as in *S. wilsonii* [Fig. 1e,f] (bottom). Colour coding shows the mutualistic strategies.

In the most species-rich ant-plant clade, the SE Asian Hydnophytinae, a subtribe of Rubiaceae with over 100 species^24–27^, each plant forms a single domatium in a swollen stem section directly below the cotyledons (hypocotyl). The domatia form regardless of the presence of ants. Species of Hydnophytinae vary in the specificity of their relationship with ants: some form generalist symbioses with essentially any arboreal ant species, others form specialized symbioses with just one or two species from one or two genera of the ant subfamily Dolichoderinae, and yet others have secondarily lost the symbiosis but retained the hypocotyl domatia, then occupied by other invertebrates and even tree frogs^25–29^. In generalist Hydnophytinae (and in species that secondarily lost ant mutualists), the domatium grows diffusely. In the specialized species, however, the domatium grows apically. The latter species usually have single stems that become thicker as their domatium grows, while the former species have much-branched slender stems^24,30–33^ (Figs 1, 2). The correlated growth of stem and domatium in species with single stems and apically growing domatium (Fig. 1a,b) could affect leaf/stem scaling, but the lack of stem/domatium ontogenetic correlation in multi-stemmed species (Fig. 1c–f) suggests that domatium growth should not influence stem diameter in multi-stemmed species.

Their different architectures (single stem, multiple stems) and domatium growth types (apical, diffuse) make the Hydnophytinae a good system in which to test how traits selected for by symbionts (i.e., domatium size or growth type) have affected evolutionary allometry. Because leaf size correlates with climate^34,35^, studies of evolutionary stem/leaf allometry should also consider trait correlation with climate variables. We therefore incorporated species’ climate niches into the analyses. Our expectation was that domatium growth in the hypocotyl (not the stem), under selection from ant symbionts, might influence stem development, while climate (annual temperature and precipitation regimes) might independently select on leaf size. We also tested a new approach for studying evolutionary allometry in a phylogenetic framework, namely the use of residuals from phylogenetically-controlled allometric regressions, which provides a species-specific scaling parameter and allows to test for shifts in allometry along the phylogeny. As far as we are aware, this is the first use of this approach to study evolutionary allometry.

## Materials and Methods

### Phylogeny of the Hydnophytinae and ancestral state reconstructions

We used a six-marker phylogeny for the Hydnophytinae from Chomicki and Renner^27^ sampling 70 of their ~104 species plus 11 outgroups. Vouchers, geographical origin and GenBank accession numbers for all sequences are reported in our earlier paper^27^. To infer the ancestral architecture type in Hydnophytinae, we coded architecture for each species as multiple-stemmed, non-domatium correlated (‘0’) or single-stemmed, correlated with domatium (‘1’), based on literature^25–28,30–33^ and our personal observations; Figs 1, 2 show the types of Hydnophytinae architecture. Note that old individuals in a few *Myrmecodia* species (e.g., *M. beccarii*) may have several stems formed by a process called reiteration (see Chomicki *et al*.^36^). To infer the evolution of domatium growth type, we coded species with domatia growing diffusely as ‘0’ and domatia growing only apically as ‘1’, using data from Jebb^37^ and the first author’s observations in the field, living collections, and in herbarium specimens.

Ancestral-state reconstructions relied on the Maximum Clade Credibility (MCC) tree obtained in Chomicki and Renner^27^ and either (*i*) used the stochastic mapping approach of Bollback^38^ implemented in the R package phytools v. 04–60^39^ (function ‘make.simmap’) or (*ii*) took phylogenetic uncertainty into account by using the Bayesian reverse-jump Markov Chain Monte Carlo (MCMC) approach implemented in BayesTraits v. 2^40^. We estimated ancestral states under the equal rates (ER) model (selected as best fitting our dataset using the Akaike Information Criterion, AIC) and then simulated 1,000 character-histories on the MCC tree. We summarized the 1,000 simulated histories using the function ‘describe.simmap’. For the reverse-jump approach we used a sample of 1,000 trees from the molecular clock analysis of Chomicki and Renner^27^ and a chain of 50 million generations and sampled rate coefficients and ancestral states every 1,000^th^ generation. We ensured that the acceptance rate was between 20 and 40%, as recommended in the manual, and reconstructed the nodes of interest using the command ‘addnode’. We reconstructed all key nodes and reported the probabilities above nodes in Fig. 2.

We tested if architecture (single stem, multi-stemmed) evolved jointly with domatium growth type using Pagel’s method in BayesTraits and the same MCC tree as above (but with the outgroups pruned). We first ran a model of independent trait evolution and estimated the four-transition rate parameters α1, α2, β1, β2, wherein double transitions from state 0, 0 to 1, 1 or from 0, 1 to 1, 0 are set to zero. We then ran a model of dependent trait evolution with eight parameters (q12, q13, q21, q24, q31, q34, q42, q43). To compare these non-nested models, we used Bayes Factor scores.

### Measurements of leaf, stem, and domatium size

We first characterized architectural types by collecting measurements of stem and domatium diameter throughout ontogeny for three species that exemplify the basic types of architecture found in the Hydnophytinae. Measurements were taken in the field (*Squamellaria wilkinsonii* and *S. wilsonii*) or in a greenhouse in Munich (*Myrmecodia tuberosa*).

Using the software imagej (http://rsb.info.nih.gov/ij), we then measured leaf area and stem cross-sectional area in 286 herbarium specimens, including 16 of *Anthorrhiza*, 128 of *Hydnophytum*, 97 of *Myrmecodia*, 10 of *Myrmephytum*, 24 of *Squamellaria* and 11 appropriate outgroups (Supplementary Dataset S1). On these specimens, we measured the area (leaves) and diameter (stems) of 115 leaves and 23 stems for *Anthorrhiza*, 1346 leaves and 453 stems for *Hydnophytum*, 527 leaves and 101 stems for *Myrmecodia*, 64 leaves and 12 stems for *Myrmephytum*, 281 leaves and 62 stems for *Squamellaria*, and 75 leaves and 13 stems for the outgroups. All specimens were mature plants, and we measured only fully expanded leaves and mature primary stems. Stem cross-sectional area was calculated as π.r^2^, where r is the radius of the stem, assuming that stem cross sections are circular. Hydnophytinae have no endogenous (or seasonally induced) growth cessation (instead they have continuous growth, cf. ref.^36^), and we therefore related the area of fully expanded leaves to the cross-sectional area of the stem where its primary growth has terminated. Termination of primary growth was determined by checking if the regular increase in shoot diameter with each new internode had stopped^41^. For much-branched species (such as most *Hydnophytum* species), this point corresponded to approximately the middle between a stem’s base (where it is attached to the domatium) and its apex. For single-stemmed species with simultaneous domatium-stem growth, it was sometimes closer to the apex (between 50% and 75% of the shoot’s length from base to apex). For each stem measured, we strove to measure at least *N* = 10 leaves, although this was not always possible. All measurements are provided as Dataset S1.

### Continuous trait fitting

Leaf and stem cross-sectional area were log-transformed for all analyses. To determine which continuous trait model best fitted our datasets (using AIC), we relied on the R package Geiger^42^. We tested all continuous trait models provided in the Geiger package (Brownian Motion [BM], Ornstein–Uhlenbeck [OU], Early Burst [EB], trend, lambda, kappa, delta, drift, white). The OU process can be conceptualized as particles under Brownian motion under friction. Thus, it models effectively the signatures of directional selection, which could be expected in our case if, as we predict, leaf and stem sizes are under divergent and distinct selection pressures. The OU process, which can model a changing adaptive landscape over time and lineages, fit our dataset best.

### Characterizing differences in intercept and slope of leaf/stem PGLS regressions among species

Based on all leaf and stem measurements (Supplementary Dataset S1), we calculated the average leaf area and leaf cross-sectional area (log-normalized) for each species to conduct phylogenetically controlled analyses. This was required to analyse the data in a phylogenetic framework, and it allowed us to minimize the effect of within-species variation in our analyses. To probe the scaling relationships between leaf and stem sizes across species we relied on the Phylogenetic Generalized Least Square (PGLS) approach using the R packages nlme^43^, phylolm^44^ and Geiger^42^. PGLS is a phylogenetic comparative method that allows testing for a correlation between two continuous variables, taking into account that lineages are not independent but linked by phylogenetic history. Related species are expected to have similar traits because of shared ancestry, and PGLS uses phylogenetic variance-covariance matrices to remove phylogenetic autocorrelation. We relied on the nlme function ‘gls’, assuming that the error structure follows an OU model (model best fitting our leaf and stem datasets, Table S1).

### Using PGLS residuals in comparative phylogenetic analyses

To infer how leaf/stem scaling evolved along the phylogeny, we wanted to have a species-specific measure of evolutionary allometry that could be used in comparative analyses. One way to achieve this would be to obtain slope and intercepts for each species and then use this in a comparative-phylogenetic context. This would inform static allometry (within-species [same stage] variability of scaling), while we were interested in evolutionary allometry. We thus decided to use residuals as a measure of scaling, since they provide species-specific information about leaf and stem sizes. Because each point (species) can be close or far from the fitted regression, the distance between each point and the regression slope (residual) encapsulates information about the scaling relationship (in our case leaf/stem scaling) of each species. We again used the OU process (best fitting our dataset) and the function ‘OUshift’ in the R package ‘phylolm’^44^ and a maximum of ten shifts allowed (nmax = 10), using an AIC framework for model selection. To quantify the variance in leaf and stem sizes across species, we used the residuals from our previous PGLS analyses (Table 1), retrieved using the function ‘residuals’ in phylolm.

**Table 1:** PGLS analysis of intercept and slope of mutualistic strategies and plant architecture.

Group	Slope	Standard error	*t*-value	*P*-value	Intercept	Standard error	*t*-value	*P*-value
*Plant architecture*								
Multi-stemmed	**0.54**	0.05	9.29	0	**−4.12**	0.66	**−**6.15	0
Single-stemmed	**0.39**	0.33	1.19	0.24	**0.47**	1.40	0.33	0.73
*Mutualistic strategy*								
Specialized	**1.00**	0.22	4.58	1E-04	**−2.88**	1.11	**−**2.60	0.01
Generalist	**0.77**	0.08	9.81	0	**−4.69**	0.55	**−**8.48	0
Non-ant associated	**0.49**	0.07	6.57	0	**−4.18**	0.44	**−**9.43	0
No domatia (outgroups)	**0.59**	0.10	5.81	3E-04	**−5.24**	0.86	**−**6.11	2E-04

Because PGLS residuals are not phylogenetically independent^45^, we used a phylogenetic ANOVA to test the significance of PGLS residuals in the different groups, using the function ‘phylANOVA’ in the R package phytools, using 10,000 simulations as recommended to obtain stable *p*-values and post-hoc tests to evaluate the significance among all pairs of groups. This analysis allowed to test whether our leaf/stem residuals varied significantly between mutualistic strategies or growth forms, while controlling for phylogenetic autocorrelation.

For purposes of visualization, we used phenograms (function ‘phenogram’ in phytools) to plot residuals against time-calibrated phylogenies, with the ancestral histories shown for our groups of interest (growth form or domatium type and mutualistic strategy [specialized interaction just with one or two species of ants vs. generalist interactions with many species of ants]).

### Testing the correlation between leaf/stem scaling and climate

Shifts in leaf/stem scaling could arise from the result of selection by ants, but it could also reflect climate variables acting on leaves (less intuitively on stems). We therefore calculated correlations between climate variables and (i) leaf/stem scaling [PGLS residuals], (ii) leaf area, and (iii) stem cross-section area. We used the 19 bioclimatic variables from the CHELSA global model (version 1.2)^46,47^. We used a dataset of 4289 georeferenced Hydnophytinae occurrences (plus appropriate outgroups) gathered from cleaned Global Biodiversity Information Facility (GBIF) (https://www.gbif.org) data and manually added herbarium specimens seen by the first author for the Hydnophytinae, and from cleaned GBIF data for the outgroups. The cleaning consisted in removing duplicates and obvious errors (such as 0,0 coordinates), or erroneous reports of the occurrence, for instance when the herbarium location is recorded instead of the collection place. We used the R package ‘raster’^48^ to extract all 19 CHELSA Bioclim variables (http://chelsa-climate.org/bioclim/). The dataset with all 4289 georeferenced data points and associated CHELSA Bioclim values is provided as Supplementary Dataset S2.

To test for correlations between climate variables and our three traits of interest while controlling for phylogenetic autocorrelation, we used the MCMC random walk model (model A) for continuous characters implemented in BayesTraits v. 3 (http://www.evolution.rdg.ac.uk/BayesTraitsV3/BayesTraitsV3.html) through R using the wrapper package “btw”^49^. We first ran a correlated model and then a model where the correlation was set to zero using the command ‘TestCorrel’. For each correlation, we evaluated correlated and non-correlated models under MCMC, estimating the log marginal likelihood using the stepping stone (SS) method^50^ with 100 stones and 1,000 iterations per stone. We compared these non-nested models, using Bayes Factor (BF) scores. Bayes Factor scores were calculated as twice the difference of marginal likelihoods, Log(BF) = 2 * (SS_corr_ − SS_non-corr_), where SS_corr_ and SS_non-cor_ are the marginal likelihoods –estimated using the stepping-stone method– of the correlated models and the model where the correlation has been set to zero, respectively.

Controlling for phylogenetic autocorrelation required using one value per species, hence we used (i) the mean, (ii) the 5% quantile, and (iii) the 95% quantile of CHELSA Bioclim values. This allowed us to better account for the range of intraspecific variation in our dataset. We ran this BayesTraits correlation model for each of the three traits (leaf/stem scaling [PGLS residuals], leaf area, and stem cross-section area), each of the 19 CHELSA Bioclim variables, and for the mean, 5% and 95% quantiles, all under correlated and non-correlated models, so a total of 342 BayesTraits runs, for a total of 171 trait-climate pairs.

We sought to identify climatic variables affecting leaf/stem allometry. To do so, we focused on climatic variables that would correlate with either leaf area or stem cross-section area, but not both. We did not consider correlates of the leaf/stem allometry itself, because correlation with a proxy (here PGLS residuals) may reflect a numeric artefact rather than a meaningful biological relationship. To visualize how leaf area versus stem cross-section area correlated with two Bioclim variables of interest (bio 15: Precipitation Seasonality and bio 19: Precipitation of Coldest Quarter, see *Results*), we performed Phylogenetic Generalized Least Square (PGLS) analyses as described above. Hydnophytinae occur in a variety of tropical environments from mangroves and lowland tropical rainforests to dry forests in Australia and New Guinea and alpine environments in New Guinea^27^, and hence species are likely to experience climate variation, notably differences in precipitation.

## Results

### Leaf/stem scaling in the Hydnophytinae

The data obtained here reveal that in species of Hydnophytinae with a single stem and an apically growing domatium, stem growth is tightly correlated with domatium growth across ontogeny (Fig. 1a,b). By contrast, in multi-stemmed species, stem growth is decoupled from domatium growth, regardless of whether domatium development is diffuse or apical (Fig. 1c–f). Diffuse domatium growth is exemplified by *Squamellaria wilkinsonii* (Fig. 1c,d), apical domatium growth by *S. wilsonii* (Fig. 1e,f). Ancestral state estimations for stem architecture (branching mode) and domatium growth further showed that species with single-stemmed architectures evolved three times from ancestors with multi-stemmed ones (Fig. 2a), and that species with apical domatium growth evolved four times from ancestors with diffusely-growing domatia (Fig. 2b). Architecture and domatium growth type were statistically correlated (Bayes Factor = 33.69).

**Figure 3:**
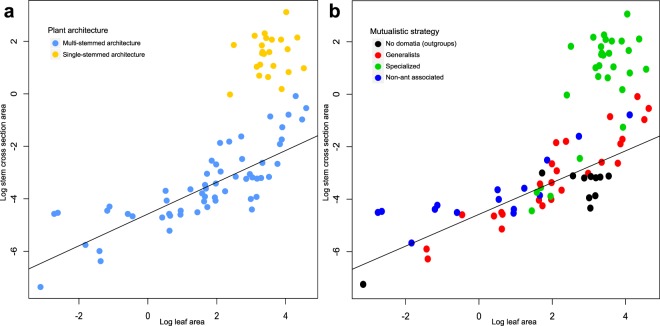
Phylogenetic Generalized Least Square (PGLS) regression of log stem cross-sectional area on log leaf area in the Hydnophytinae, fitting an Ornstein-Uhlenbeck model. The PGLS regression for the whole dataset is y = 0.60x–4.58 (Intercept: F_1,79_ = 12.27, *P* < 0.001; slope: F_1,79_ = 93.53, *P* < 0.001). Plant architecture is shown in (a) and mutualistic strategy in (b). Each dot represents a distinct species sampled in the Hydnophytinae phylogeny.

In all cases, the OU process model best fit the log-normalized leaf area and stem cross-sectional area datasets (Table S1, which also shows model parameters and AIC values). The PGLS analysis of Log Stem cross section area on Log Leaf area revealed strong variability in leaf/stem proportions (Fig. 3a,b) and showed that single-stemmed species had a much larger intercept (0.47 ± 1.4) than multi-stemmed species (−4.12 ± 0.66; Table 1; Fig. 3).

### Evolution of allometry inferred in a phylogenetic framework using PGLS residuals

**Figure 4:**
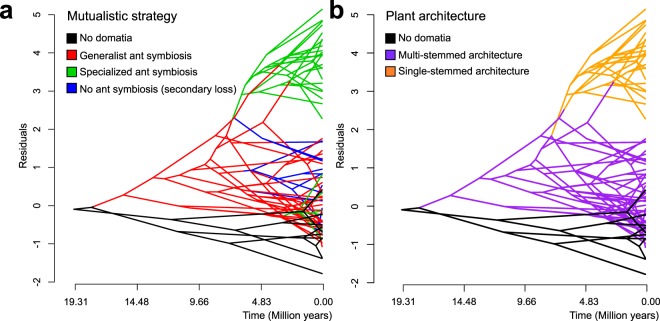
Phenograms showing the Hydnophytinae phylogeny plotted against PGLS residuals and time for mutualistic strategies (**a**) and plant architecture (**b**). The clustering of the majority of specialized ant-plants corresponds to the three (out of four) species groups or species with single-stemmed architecture (which all have apically growing domatia; cf. Figs 1, 2).

We retrieved the residuals (each representing a different species) from the PGLS analysis and performed phylogenetic ANOVAs to test how they varied among four groups, namely species forming specialized symbiosis with one or two ant species; species forming generalist symbiosis with any arboreal ant; species having lost the symbiosis; and outgroups lacking domatia (*Introduction*). The distribution of these residuals revealed a clear departure from the scaling relationship (Fig. 3), and residuals differed significantly among the groups (F = 39.12, *P* = 0.004). *Post hoc* comparisons indicated that domatium-bearing species forming specialized symbioses were significantly different from all other groups (*post hoc* test; outgroups lacking domatia vs. species forming specialized symbioses; *P* = 0.05; species forming generalist symbioses vs. species forming specialized symbioses, *P* = 0.0012; species having lost ant symbiosis vs. specialized species, *P* = 0.002), while other contrasts were not significant. These results of our approach using residuals are congruent with the comparison of intercepts and slopes among groups (Fig. 3, Table 1). Phenograms visually revealed the difference in residuals in the different groups (Fig. 4).

**Figure 5:**
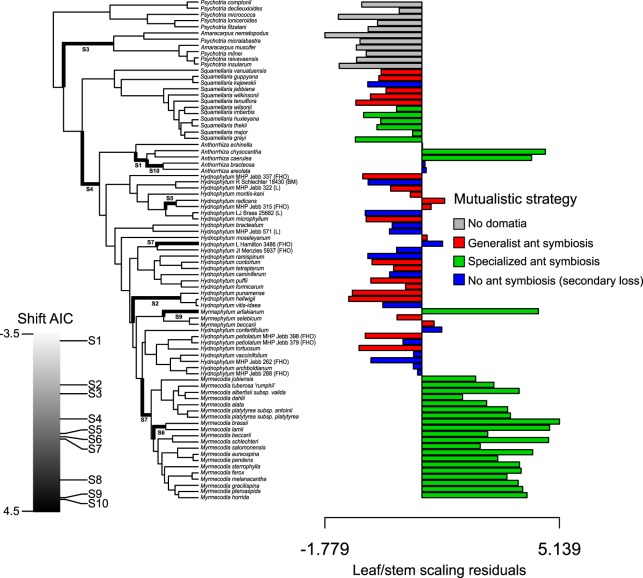
Using PGLS residuals to study evolutionary allometry in phylogenetic framework. Shifts in the Ornstein-Uhlenbeck (OU) process mirror mutualistic strategies in the Hydnophytinae. A maximum of 10 shifts were allowed, and the order of estimated shifts was determined by AIC. Shifts in the OU selection optimum μ are shown by bold branches and labelled from S1 (best) to S10 (worse) based on AIC and shown with bold branches on the tree. The three species groups or species of specialized ant-plants with unusually large residuals comprise species with single-stem architecture (Figs 3, 4).

Since the residuals yielded results congruent with the regression approach, we next used residuals to conduct phylogenetic comparative analyses. Specifically, we used PGLS residuals to ask whether there were shifts in evolutionary rate of leaf/stem scaling along the tree and if these shifts correlated with the evolution of domatium growth type (apical or diffuse). We used the OU process to model the evolution of residual rate (*Materials and Methods*). We found that 9 of 10 shifts in the OU selection optimum (thereafter “OU shifts”) occurred in the domatium-bearing clade and that most were correlated with transitions in domatium growth type (Fig. 5). We also performed the same analyses independently for (log-normalized) leaf and stem sizes. This showed that OU shifts in leaf size showed no obvious correlation with domatium growth type, while analyses of stem diameter showed similar patterns to the residuals (Fig. S1).

### Trait-climate correlated evolution

**Figure 6:**
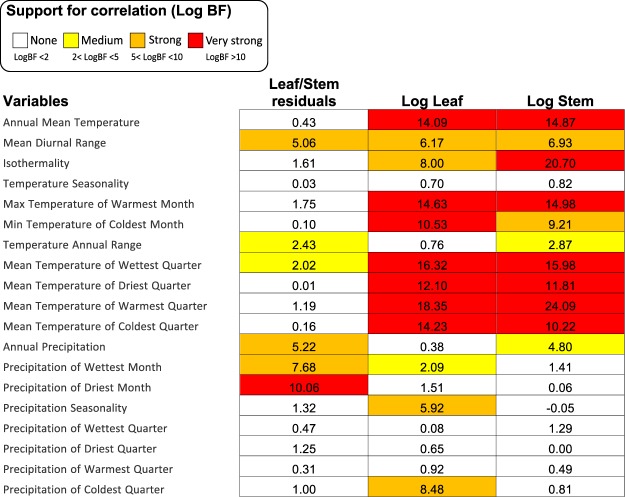
Trait-climate phylogenetic correlations (*Materials and Methods*) for each of the 19 CHELSA Bioclim variables using the 95% percentile dataset. Values show Bayes factor scores, with yellow illustrating somewhat correlated variables (2 < logBF < 5), orange strongly correlated variables (5 < logBF < 10), and fifteen were very strongly correlated variables (logBF > 10) and white cells show no correlation. See also Table S2 and S3 for trait-climate correlations with 95% and 5% percentiles CHELSA Bioclim datasets.

Of the 57 trait-climate (mean CHELSA Bioclim values) pairs, five were somewhat correlated (2 < logBF < 5), nine were strongly correlated (5 < logBF < 10), and fifteen were very strongly correlated (logBF > 10) (Fig. 6). The analyses with 5% and 95% quantiles were consistent with that of the mean Bioclim values (Tables S1, S2). Most of the correlations with climate were the same for leaf and stem sizes. We thus focused on strong correlations between climate and leaf or stem, but not both, the rationale being that a climatic variable affecting one organ but not the other could affect allometry. No strong correlation with climate was detected in stem diameter alone (Fig. 6). Leaf size, however, was strongly positively correlated with precipitation seasonality (bio 15) and precipitation of the coldest quarter (bio 19).

**Figure 7:**
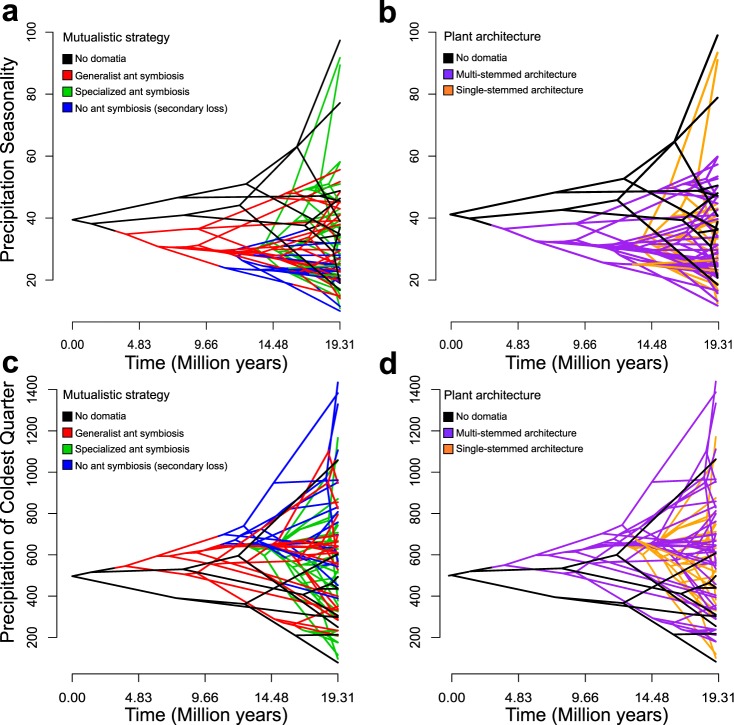
Phenograms showing the Hydnophytinae phylogeny plotted against precipitation seasonality (Bio 15) mapping mutualistic strategies (**a**) and vegetative growth architecture (**b**) on the tree, and precipitation of the coldest quarter (Bio 19) mapping mutualistic strategies (**c**) and vegetative growth architecture (**d**) on the tree.

To visualize how climate correlates with leaf size, we used phenograms. No clustering of the four mutualistic strategies (previous section) was detected for bio 19 (Precipitation of Coldest Quarter), but species that have lost symbiosis with ants have a narrower range in Precipitation Seasonality (bio 15) than do species with generalist ant symbioses and species with specialized ant symbioses (Fig. 7), matching the altitudinal range of these different mutualisms^27^.

**Figure 8:**
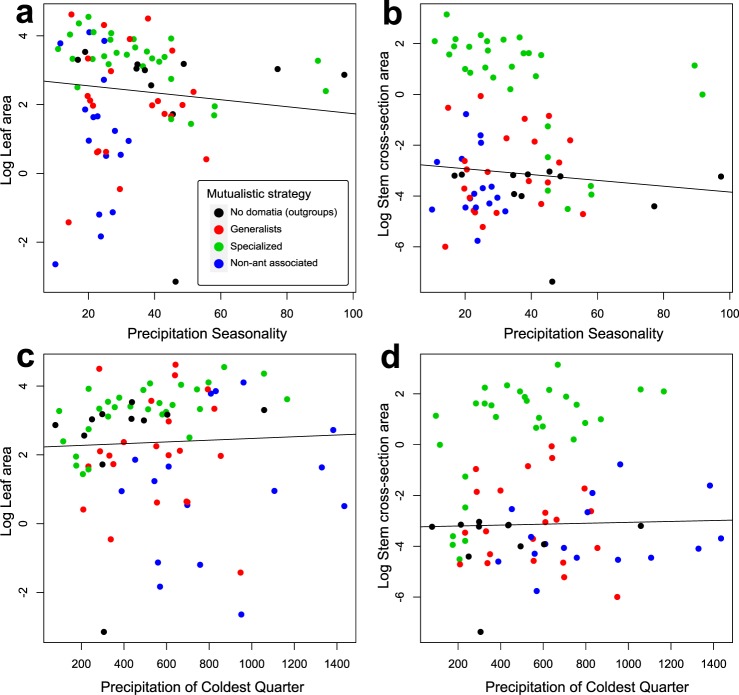
Phylogenetic Generalized Least Square (PGLS) regression of leaf area and stem cross-section area on precipitation seasonality and precipitation of the coldest quarter. (**a**) Leaf area *vs*. Precipitation seasonality (y = −0.01 + 2.75; Intercept: F_1,79_ = 2.08, *P* = 0.15; Slope: F_1,79_ = 0.71, *P* = 0.39). (**b**) Stem cross-section area *vs*. Precipitation seasonality (y = −0.01–2.69; Intercept: F_1,79_ = 5.57, *P* = 0.02; Slope: F_1,79_ = 1.37, *P* = 0.24). (**c**) Leaf area *vs*. precipitation of the coldest quarter (y = 0.0002 + 2.22; Intercept: F_1,79_ = 2.06, *P* = 0.15; Slope: F_1,79_ = 0.15, *P* = 0.69). (**d**) Stem cross-section area *vs*. precipitation of the coldest quarter (y = 0.0002–3.24; Intercept: F_1,79_ = 5.48, *P* = 0.02; Slope: F_1,79_ = 0.11, *P* = 0.76).

We next used phylogenetic generalised least square (PGLS) regressions (*Materials and Methods*) to visualize the correlations between our two Bioclim variables of interest (precipitation seasonality and precipitation of the coldest quarter) and leaf size *versus* stem cross-section area. This revealed that leaf size and stem cross-section area are independently correlated with these variables, but the regression slopes were not significant while the regression intercept was significant for stem cross-section and both precipitation variables (Fig. 8).

## Discussion

### Evolutionary shifts in leaf/stem scaling in the Hydnophytinae imply divergent selection from ant symbionts and climate

Our analyses reveal that the evolution of hypocotyl domatia in the most recent common ancestor of the Hydnophytinae and subsequent different symbiotic strategies have strongly impacted leaf/stem scaling. These scaling relationships differ between single-stemmed species in which stem primary development is correlated with apical domatium growth and multi-stemmed species with diffuse domatium growth. Shifts in leaf/stem scaling occurred at least three times (Fig. 5) and are tied to shifts in intercept, but not in slope (Table 1), suggesting that domatium presence consistently changed stem diameter but not leaf size. Our results also show that the evolution of single-stemmed architectures and apical domatium growth (in the hypocotyl) led to an increase in stem diameter.

The inferred three shifts in leaf/stem scaling appear to result from opposing selective forces. First, the combination of apical domatium growth with single stems led to correlated domatium/stem development (Fig. 1) because stem diameter increases with domatium volume (even though the domatia are not formed inside stems). In single-stemmed species the domatium constrains the stem size because of two reasons. The first is a developmental one: The stem expands concurrently with the domatium since they are developmental integrated, thus limiting phytomer size. This is different in multi-stemmed species where delayed branching uncouples phytomer development^27,36^. The second is a vascular one: The larger the domatium, the more photosynthates it requires, which in turn requires more extensive vasculature. Second, leaf size and stem cross-section area (Fig. S1) evolved independent of each other, also supporting that divergent selective pressures act on these two traits. Leaf size, but not stem cross-section area, correlates with precipitation seasonality and precipitation of the coldest quarter (Figs 6–8), in agreement with well-documented correlations of leaf size with precipitation and temperature^34,35^. In combination, these results suggest that symbiosis with ants has imposed selection on stem diameter, while precipitation has driven selection on leaf area.

The phylogenetic generalized least square (PGLS) regressions of leaf area and stem cross-section area on precipitation seasonality and precipitation of the coldest quarter also clearly showed the distinct correlations (Fig. 8). That rainfall variables have participated in the shifts in leaf/stem allometry makes sense because the (almost exclusively) epiphytic Hydnophytinae occur in a variety of tropical environments from mangroves and lowland tropical rainforests to dry forests in Australia and New Guinea and alpine environments in New Guinea^27^. In terrestrial plants, water availability can drive leaf/stem allometry^51^.

In ant-plants with hollow stem domatia, such as *Cecropia* or *Macaranga*, shifts in leaf/stem scaling are expected because the new function of the stem affects its size and biomechanical properties^21,22,52^. By contrast in Hydnophytinae, the stems (regardless of plant architecture or domatium growth type) are solid, not hollow, because the domatium is formed in the hypocotyl. Thus, stem size is only affected if its growth is correlated with that of the domatium. In hollow-stemmed ant-plants, by contrast, the domatium is costly early in development since extra wood is required to maintain mechanical stability before secondary stem growth has started^21,22,52^. The shift in leaf/stem scaling in Hydnophytinae with a single-stemmed architecture and apical domatium growth implies larger stems for the same leaf size. This could be because single-stemmed species have fewer leaves and a smaller photosynthetic capability than a multi-stemmed species, perhaps implying a carbon cost that could influence the mutualism.

### Analyzing allometry in a phylogenetic context: the use of PGLS residuals

Studies of allometric scaling in animals have focused on ontogenetic allometry, with evolutionary allometry being least studied. The same goes for plant allometry^53^. Geometric morphometric studies across taxa are increasingly mapping shape variation onto phylogenies by plotting trees onto a PCA-based morpho-space^54–56^. By contrast, phylogenetic trait regressions, such as PGLS, allow comparison of slopes and intersects^57–60^. For ontogenetic or static allometry, intercepts or slopes can directly be used as continuous traits, while residuals can provide species-specific measurements of scaling relationships and be used as continuous traits in ancestral state reconstructions to study evolutionary allometry. In this study, we used residuals from phylogenetic regressions in addition to these methods. Because we are dealing with evolutionary allometry, each data point represents a species, and only residuals can give a measure of scaling for a given species. Moreover, residuals, but not slopes and intercepts, also inform about statistical ‘noise’ in scaling relationships. However, residuals obtained from PGLS analyses are non-phylogenetically independent, and their comparison thus requires either further phylogenetic analyses (as we did here) or multiplication by the inverse of the phylogenetic variance-covariance matrix^45^.

### Mutualistic symbioses affect traits beyond those directly affected by the partners

Shifts in the level of mutualism specificity (generalist to specialized) and dependency (facultative to obligate) affect the evolutionary pace of interaction-related traits, probably through stabilizing selection^27,61,62^. Here we showed how shifts in mutualistic strategies are correlated with changes in leaf/stem scaling (not obviously under selection by ants) and domatium growth type (probably under selection by ants). The effects of species interactions on trait evolution are of great interest in evolutionary biology and ecology. Work in this area has largely focused on traits directly involved in an interaction, as they are the ones most obviously influencing species’ responses. Analysing the ‘side effects’ of symbiotic mutualisms as done here may throw light on gains and losses of symbioses, both evolutionarily and ecologically. Mutualisms with ants involve pollination (conflicts between ants and pollinators that needs to be resolved), chemical defences, leaf phenology, and leaf/stem scaling^21,22,63–65^ (this study), and they likely affect many other traits in addition to those directly involved in the respective interaction. Studies of scaling relationships in further mutualisms may contribute to better models of their costs and benefits and thereby to a better understanding of mutualism stability.

## Conclusion

This study has shown how divergent selective pressures on stems and leaves can lead to shifts in leaf/stem scaling. We have used and advocate residuals from PGLS regressions as a way to perform comparative-phylogenetic analyses of evolutionary allometry. The results highlight how symbiotic mutualisms can modify widespread allometric rules. Thus, shifts in allometry may affect costs and benefits within mutualisms, and thereby affect their ecological and evolutionary stability.

## Supplementary information

Supplementary online materials

Dataset S1

Dataset S2
