# Supplementary material for: Climate and symbioses with ants modulate leaf/stem scaling in epiphytes
Source: Sci Rep. 2019 Feb 22;9:2624. doi: 10.1038/s41598-019-39853-4 (PMC6385368; doi:10.1038/s41598-019-39853-4)
Supplement: Supplementary file 1 — Supplementary online materials [file 41598_2019_39853_MOESM1_ESM.pdf]

Supplementary information for

Climate and symbioses with ants modulate leaf/stem scaling in epiphytes

Authors: Guillaume Chomicki[1,2] and Susanne S. Renner[3]

Affiliations: [1]Department of Plant Sciences, University of Oxford, South Park Road, Oxford OX1 3RB, UK. [2]The Queen's college, High St, Oxford OX1 4AW, UK. [3]Systematic Botany and Mycology, Department of Biology, University of Munich (LMU), Menzinger Str. 67, 80638 Munich.

Correspondence: G. Chomicki (guillaume.chomicki@gmail.com)

This pdf contains:

Supplementary figure S1

Supplementary tables S1-S3

Note that the supplementary materials also include two supplementary datasets (Supplementary datasets S1 and S2), which are provided in separate excel files.

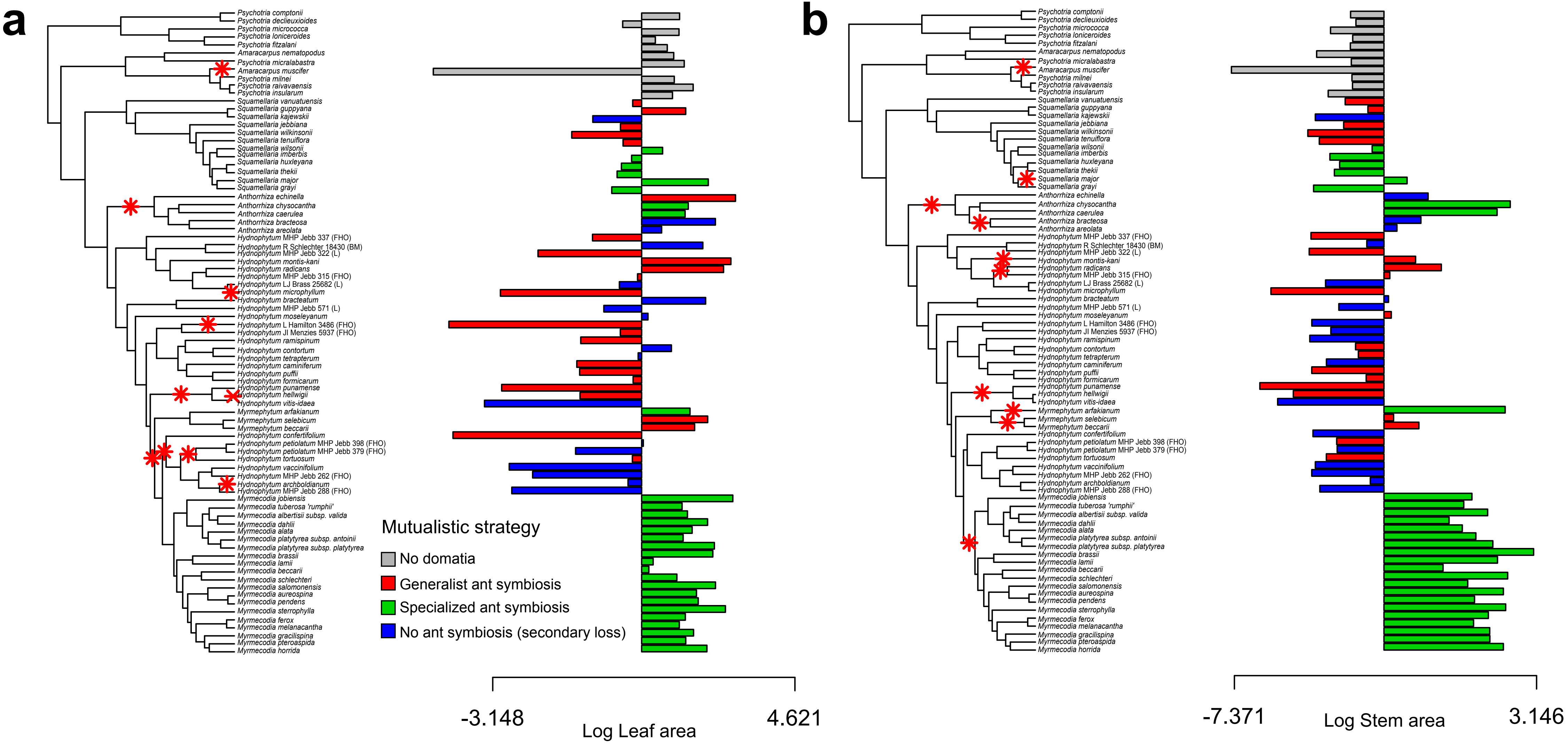

Figure S1. Shifts in the Orstein-Uhlenbeck (OU) in log leaf area (A) and log stem (B) cross-section area in the Hydnophytinae. A maximum of 10 shifts were allowed, and the order of estimated shifts were determined by AIC. Shifts in the OU selection optimum μ are shown by red stars on the tree.

Table S1. Model-testing for Log leaf area, Log stem cross-section area, and PGLS residuals for the Hydnophytinae.

HYDNOPHYTINAE	**BM**		**OU**		**EB**		**TREND**		**LAMBDA**		**KAPPA**		**DELTA**		**DRIFT**		**WHITE**	
**METRIC**	**AICc**	**DF**	**AICc**	**DF**	**AICc**	**DF**	**AICc**	**DF**	**AICc**	**DF**	**AICc**	**DF**	**AICc**	**DF**	**AICc**	**DF**	**AICc**	**DF**
**LOG LEAF**	350.51	2	326.62	3	352.67	3	343.51	3	324.86	3	333.90	3	337.28	3	352.67	3	331.05	3
**LOG STEM**	318.77	2	319.92	3	320.93	3	319.16	3	319.26	3	313.70	3	319.15	3	320.93	3	388.3	3
**PGLS RESIDUALS**	253.92	2	256.32	3	258.48	3	257.69	3	257.87	3	247.43	3	257.45	3	258.48	3	338.7	3

Table S2. Trait-climate phylogenetic correlations (*Materials and Methods*) for each of the 19 CHEALSA bioclim variables using the 95% percentile dataset. Values show Bayes factor scores, with yellow illustrating somewhat correlated variables (2<logBF<5), orange strongly correlated variables (5<logBF<10), and fifteen were very strongly correlated variables (logBF> 10) and white cells show no correlation.

Variables	Leaf/Stem residuals	LN_Leaf	LN_Stem
bio1_0.95	2.82	14.09	14.87
bio2_0.95	-0.10	6.17	6.93
bio3_0.95	0.15	8.00	20.70
bio4_0.95	-0.01	0.70	0.82
bio5_0.95	3.04	14.63	14.98
bio6_0.95	4.04	10.53	9.21
bio7_0.95	0.33	0.76	2.87
bio8_0.95	2.22	16.32	15.98
bio9_0.95	4.26	12.10	11.81
bio10_0.95	3.25	18.35	24.09
bio11_0.95	2.75	14.23	10.22
bio12_0.95	8.55	0.38	4.80
bio13_0.95	11.87	2.09	1.41
bio14_0.95	5.68	1.51	0.06
bio15_0.95	0.46	5.92	-0.05
bio16_0.95	17.54	0.08	1.29
bio17_0.95	1.45	0.65	0.00
bio18_0.95	1.52	0.92	0.49
bio19_0.95	12.52	8.48	0.81

Table S3. Trait-climate phylogenetic correlations (*Materials and Methods*) for each of the 19 CHEALSA bioclim variables using the 5% percentile dataset. Values show Bayes factor scores, with yellow illustrating somewhat correlated variables (2<logBF<5), orange strongly correlated variables (5<logBF<10), and fifteen were very strongly correlated variables (logBF> 10), and white cells show no correlation.

Variables	Leaf/Stem residuals	LN_Leaf	LN_Stem
bio1_0.05	1,93	14,09	14,87
bio2_0.05	3,84	6,17	6,93
bio3_0.05	8,04	8,00	20,70
bio4_0.05	2,27	0,70	0,82
bio5_0.05	8,59	14,63	14,98
bio6_0.05	1,89	10,53	9,21
bio7_0.05	4,89	0,76	2,87
bio8_0.05	3,55	16,32	15,98
bio9_0.05	2,95	12,10	11,81
bio10_0.05	3,41	18,35	24,09
bio11_0.05	3,50	14,23	10,22
bio12_0.05	0,58	0,38	4,80
bio13_0.05	0,13	2,09	1,41
bio14_0.05	0,05	1,51	0,06
bio15_0.05	0,56	5,92	-0,05
bio16_0.05	1,09	0,08	1,29
bio17_0.05	0,51	0,65	0,00
bio18_0.05	2,03	0,92	0,49
bio19_0.05	0,08	8,48	0,81
